# Force-Sensorless Identification and Classification of Tissue Biomechanical Parameters for Robot-Assisted Palpation

**DOI:** 10.3390/s22228670

**Published:** 2022-11-10

**Authors:** Alejandro Gutierrez-Giles, Miguel A. Padilla-Castañeda, Luis Alvarez-Icaza, Enoch Gutierrez-Herrera

**Affiliations:** 1 Centro de Estudios en Computación Avanzada (CECAv), Universidad Nacional Autónoma de México (UNAM), Mexico City 04510, Mexico; 2 Instituto de Ciencias Aplicadas y Tecnología (ICAT), Universidad Nacional Autónoma de México (UNAM), Mexico City 04510, Mexico; 3 Instituto de Ingeniería (II), Universidad Nacional Autónoma de México (UNAM), Mexico City 04510, Mexico

**Keywords:** robotics, palpation, sensorless, biomedical, force sensors, force control, estimation

## Abstract

The implementation of robotic systems for minimally invasive surgery and medical procedures is an active topic of research in recent years. One of the most common procedures is the palpation of soft tissues to identify their mechanical characteristics. In particular, it is very useful to identify the tissue’s stiffness or equivalently its elasticity coefficient. However, this identification relies on the existence of a force sensor or a tactile sensor mounted at the tip of the robot, as well as on measuring the robot velocity. For some applications it would be desirable to identify the biomechanical characteristics of soft tissues without the need for a force/tactile nor velocity sensors. An estimation of such quantities can be obtained by a model-based state observer for which the inputs are only the robot joint positions and its commanded joint torques. The estimated velocities and forces can then be employed for closed-loop force control, force reflection, and mechanical parameters estimation. In this work, a closed-loop force control is proposed based on the estimated contact forces to avoid any tissue damage. Then, the information from the estimated forces and velocities is used in a least squares estimator of the mechanical parameters. Moreover, the estimated biomechanical parameters are employed in a Bayesian classifier to provide further help for the physician to make a diagnosis. We have found that a combination of the parameters of both linear and nonlinear viscoelastic models provide better classification results: 0% misclassifications against 50% when using a linear model, and 3.12% when using only a nonlinear model, for the case in which the samples have very similar mechanical properties.

## 1. Introduction

There have been many efforts over the last few decades to incorporate robotic capabilities for improving medical procedures such as diagnostics, surgeries, and examination. An important examination procedure consists of the palpation of tissues to detect abnormalities or differences with respect to healthy tissues. In this context, several robotic systems that can potentially help practitioners to carry out a palpation task have been proposed in the literature. For example, as early as 1998, in [1] a tendon-actuated finger equipped with a tactile sensor is designed for minimally invasive data gathering during palpation procedures. Later, in [2], an integration of tactile and force sensors is proposed to enhance the location of tumors by providing force reflection to the physicians in a master–slave teleoperation scheme. In the same context, in [3], a magnified force reflection scheme is proposed to improve the user’s capabilities for performing palpation and needle insertion tasks. This same topic is studied aiming at the inclusion of force feedback in the *da Vinci Research Kit* for different tasks in a master–slave teleoperation system. In [4], the modulation of the forces applied to the tissues is investigated. The authors have found that humans tend to impose a force pattern that is independent of the non-homogeneity of the tissue. Gaussian processes are studied in [5] to construct a stiffness map to detect tissue abnormalities, while simultaneously avoiding obstacles. A combination of image processing and a miniature force sensor is proposed in [6] also to construct a stiffness map in a virtual reality application designed for its use in the *da Vinci Research Kit*. In [7], a recurrent neural network is employed to detect the depth of tumors from position and force measurements. A system that does not employ force sensors at the tip of the probe is proposed in [8], using a cable-driven mechanism capable of detecting changes on the stiffness of the tissues.

The Bayesian framework has been successfully employed in the literature for tumor detection. For example, in [9] it is employed to detect tumor inclusions at different depths by employing an industrial manipulator with a capacitive tactile sensor mounted at the tip. A similar work [10] employs a Bayesian optimization algorithm, and it is capable of detecting the boundary of a tumor within 30 iterations as well as the depth of the inclusion, based on tactile sensor measurements. When employing images for tumor detection, there are several recently obtained results. In [11], MRI images are processed to obtain higher order statistical features to detect brain tumor. In another work [12], a Bayesian fuzzy clustering algorithm is combined with a neural network for tumor detection and classification based on MRI images. The tumor growth estimation is investigated in [13] from T2-FLAIR images by employing a Bayesian inference framework. In [14], a new capsule networks are employed in a Bayesian framework to improve the detection and classification of tumors by including the knowledge of experts neurosurgeons for the uncertain prediction cases.

In this paper, we propose to use a state observer to simultaneously estimate contact forces and robot velocities for palpation tasks by employing only position measurements and the open loop joint torques commanded to the robot actuators. Thus, the observer is based on the robot dynamic model, the control inputs and the joint position measurements. Although different force and velocity observers have been proposed in the literature in the last few years [15,16,17,18], in this work we use the extended-state finite-time observer (FTO) proposed in [19], which has the advantage of providing finite time convergence of the forces and velocities to the real ones. Additionally, this observer has the advantage of its easy implementation and tuning. An accurate dynamic model of the manipulator is required to obtain a good approximation of the forces (this is not the case for the velocitites estimation, which is independent of the model accuracy). Nevertheless, to the best of the authors’ knowledge, there is no velocity and force observer independent of the model accuracy. The estimated velocity and force signals are, in turn, employed to estimate the biomechanical parameters of the palpated tissue, given an a priori specified model. An important feature of the proposed work is its capability of not only distinguish between tissues with different stiffness, but also providing an estimation of the tissue model parameters. These parameters can be employed in a number of applications, e.g., realistic simulation of the tissues in virtual reality systems [20,21] for educational/training purposes, mathematical analysis of interaction controllers, among others. A Bayesian classifier is also proposed in this paper to automatically detect the tissue which with the robot is interacting, after a training phase. Two models are proposed to model the tissue mechanics, one considering a linear elasticity and another considering a nonlinear elasticity. We have found that by combining the estimated parameters of the two models, the classification is greatly improved. In contrast with the result of [8], instead of an ad-hoc system, a standard robotic manipulator model is employed in this work, which can be an important feature towards its implementation in a real scenario. In accordance with [4], the modulation of forces is very important for the correct detection of tissue abnormalities. For such reason, a closed-loop force controller based on the estimated signals is proposed in this work, with a carefully selected force profile which permits to estimate the mechanical parameters accurately, while preventing any damage to the tissue. Furthermore, the shortest possible experimental duration time was also tuned to reduce the stress on the tissue.

The localization of the zones of interest (e.g., tumors) is out of the scope of this paper. In turn, we assume that either the physician has already identified the zone in which they want to learn its mechanical properties, or one of the several automatic algorithms reported in the literature has been implemented in a previous step, e.g., tactile sensor-based [2,9,10], force sensor (in the present work, the estimated force can be used instead of the force measured by the sensor) feedback [3,5,22], preoperative images processing [6], magnetic fields [23], MRI scans, and CT scans. A recent review on the methods for identification of zones based on medical images analysis and artificial intelligence can be found in [24].

The estimated contact force can be utilized for force-reflection, without further modifications, in a master–slave teleoperation system similar to the one proposed in [2] for tumor localization, with the advantage of obviating the force sensor requirement.

In summary, the main contributions of this article are the following:

- Simultaneous estimation of robot end-effector forces and velocities, using only joint position sensors and commanded torques.
- Estimation of tissues’ biomechanical parameters based on the estimated forces and velocities.
- A standard robot manipulator model is employed instead of an ad hoc system.
- Classification of tissues is based on the estimated parameters taking into account a linear model, a nonlinear model, and the combination of both models, giving, as a result, a better classification for the last case.

The remainder of this paper is organized as follows: In Section 2, the description of the theoretical tools to support the proposed method is presented. In Section 3, experimental results, which serve to validate the approach, are shown. A discussion section on these experimental results is presented in Section 4. Some concluding remarks and directions for future work are stated in Section 5.

## 2. Materials and Methods

### 2.1. Robot and Environment Model

In order to estimate the contact forces and the robot velocities, consider an *n*–degrees of freedom manipulator in contact with its environment. A mathematical model describing the robot motion and contact interaction is given by [25] (p. 11)

(1) $$\begin{array}{c} H\left(q\right)\ddot{q}+C\left(q,\dot{q}\right)\dot{q}+\tau_{f}\left(\dot{q}\right)+g\left(q\right)=\tau -\tau_{e}\;, \end{array}$$

where $q\in R^{n}$ is the vector of joint coordinates, $H\left(q\right)\in R^{n\times n}$ is the inertia matrix, $C\left(q,\dot{q}\right)\dot{q}\in R^{n}$ is the vector of Coriolis and centrifugal torques, $\tau_{f}\left(\dot{q}\right)\in R^{n}$ is the vector of friction effects at the joints, $g\left(q\right)\in R^{n}$ is the vector of gravitational torques, $\tau \in R^{n}$ is the vector of input torques acting at the joints, and $\tau_{e}\in R^{n}$ represents the external forces arising from the interaction with the environment. This last term, $\tau_{e}$, is a mapping from forces and torques resulting from the contact of the robot’s end-effector with the environment, given by $F_{e}\in R^{m}$, where m=6 if forces and moments are considered, and m=3 if only forces are taken into account. Thus, $F_{e}$ and $\tau_{e}$ are related by [26] (p. 148)

(2) $$\begin{array}{c} \tau_{e}=J^{T}\left(q\right)F_{e}\;, \end{array}$$

with $J\left(q\right)\in R^{m\times n}$ the Jacobian of the manipulator. Notice that such model is general, in the sense that the contact force $F_{e}$ in (1) and (2) can be any external force. For example, if a linear spring–damper mechanical system in a unidirectional axis is considered as the environment, then $F_{e}\in R^{3}$, and a mathematical model for this force is given by

(3) $$\begin{array}{c} F_{e}=k_{l}\left(x-x_{0}\right)+b_{l}v\;, \end{array}$$

where $x\in R^{3}$ is the linear position of the end-effector which can be obtained by direct kinematics through a function $x=f_{\mathrm{dk}}\left(q\right)$, $x_{0}$ is the point where the robot starts the contact with the environment without deformation, $v≜\dot{x}$ is the end-effector linear velocity, and $k_{l}$ and $b_{l}$ are the constant stiffness and damping coefficients, respectively. Alternatively, a nonlinear spring and a damper mechanical system can be considered as

(4) $$\begin{array}{c} F_{e}=k_{\mathrm{nl}}{\left(x-x_{0}\right)}^{3}+b_{\mathrm{nl}}v\;, \end{array}$$

with $k_{\mathrm{nl}}$ and $b_{\mathrm{nl}}$, the constant nonlinear-spring and damping coefficients, respectively. It has been claimed that the nonlinear cubic-spring viscoelastic models are better to represent biological tissues than the linear spring models [27,28].

In order to obtain an online estimation of both the external forces and the manipulator’s velocities, consider the states defined as $q_{1}≜q$ and $q_{2}≜\dot{q}$. The corresponding state-space representation of model (1) is

(5) $$\begin{array}{cc} {\dot{q}}_{1}= & q_{2} \end{array}$$

(6) $$\begin{array}{cc} {\dot{q}}_{2}= & H{\left(q_{1}\right)}^{-1}\left(\tau -C\left(q_{1},q_{2}\right)q_{2}-\tau_{f}\left(q_{2}\right)-g\left(q_{1}\right)\right)-H{\left(q_{1}\right)}^{-1}\tau_{e}\;. \end{array}$$

The following property guarantees that the state-space model above is always well-posed.

**Property** **1** ([29] (p. 171))**.** *The inertia matrix $H\left(q\right)\in R^{n\times n}$ is symmetric and positive definite for all $q\in R^{n}$.*

To estimate the external force, regardless of its origin, the following assumption is made.

**Assumption** **1.** *The vector of external forces $F_{e}$ is bounded.*

### 2.2. Velocity and Force Observer

By taking into account the state-space model (5) and (6) and Assumption 1, the Finite-Time Observer (FTO) proposed in [19] can be employed to simultaneously estimate the robot velocity and the external forces

(7) $$\begin{array}{cc} {\tilde{q}}_{1}= & q_{1}-{\hat{q}}_{1} \end{array}$$

(8) $$\begin{array}{cc} {\dot{\hat{q}}}_{1}= & {\hat{q}}_{2}+K_{1}{\left\lfloor {\tilde{q}}_{1}\right\rceil }^{3/4} \end{array}$$

(9) $$\begin{array}{cc} {\dot{\hat{q}}}_{2}= & H{\left(q\right)}^{-1}\left(\tau -C\left({\hat{q}}_{1},{\hat{q}}_{2}\right){\hat{q}}_{2}-\tau_{f}\left({\hat{q}}_{2}\right)-g\left({\hat{q}}_{1}\right)\right)+{\hat{\zeta }}_{1}+K_{2}{\left\lfloor {\tilde{q}}_{1}\right\rceil }^{1/2} \end{array}$$

(10) $$\begin{array}{cc} {\dot{\hat{\zeta }}}_{1}= & K_{3}{\left\lfloor {\tilde{q}}_{1}\right\rceil }^{1/4} \end{array}$$

(11) $$\begin{array}{cc} {\hat{\tau }}_{e}= & H\left(q_{1}\right){\hat{\zeta }}_{1}\;, \end{array}$$

where ${\hat{q}}_{1}$ and ${\hat{q}}_{2}$ are the estimates of $q_{1}$ and $q_{2}$, respectively, ${\hat{\zeta }}_{1}\in R^{n}$ is a new state employed for the external force estimation, the function ${\left\lfloor z\right\rceil }^{\alpha }$ is defined component-wise as

(12) $$\begin{array}{c} {\left\lfloor z\right\rceil }_{i}^{\alpha }={\left|z_{i}\right|}^{\alpha }\;\mathrm{sign}\left(z_{i}\right)\;, \end{array}$$

$K_{1},K_{2},K_{3}\in R^{n\times n}$ are diagonal gain matrices to be tuned, and α∈[0,1). In accordance with the analysis presented in [19], the FTO guarantees finite time convergence of the estimated quantities $\left({\hat{q}}_{1},{\hat{q}}_{2},{\hat{\tau }}_{e}\right)$ to the real ones $\left(q_{1},q_{2},\tau_{e}\right)$ for any positive definite matrices $K_{1}$, $K_{2}$, and $K_{3}$, but it is stated that a good result is obtained if the observer is tuned as an Extended State Observer (ESO), i.e., by considering a linear observer (with all exponents equal to one) only for tuning purposes. Notice that an accurate manipulator model is mandatory to obtain reliable estimates of the mentioned signals. Nevertheless, to the best of the authors’ knowledge, there is no force estimator for robotic manipulators independent of the model accuracy.

While the estimation of joint velocities is directly obtained through ${\hat{q}}_{2}$, the estimation of the end-effector velocities $\hat{v}\in R^{m}$ can be obtained by using the relation

(13) $$\begin{array}{c} \hat{v}=J\left(q_{1}\right){\hat{q}}_{2}\;. \end{array}$$

In turn, to obtain an estimation of the Cartesian environment forces, the following assumption must be made.

**Assumption** **2.** *The manipulator never reaches a singularity, so J(q) is always full-rank.*

Assumption 2 implies that $J^{-1}\left(q\right)$ always exists. Such an assumption can be easily fulfilled if the robot is restricted to its dexterous workspace [30] (p. 6). Therefore, an estimation of the Cartesian contact forces can be obtained through the estimation of the external torques ${\hat{\tau }}_{e}$ in (11) and the inversion of (2), i.e.,

(14) $${\hat{F}}_{e}=J^{-T}\left(q_{1}\right){\hat{\tau }}_{e}=J^{-T}\left(q\right)H\left(q_{1}\right){\hat{\zeta }}_{1}\;.$$

### 2.3. Parameter Estimation

Models (3) and (4) can be linearly parametrized as

(15) $$\begin{array}{c} \underset{Y_{l}\left(x,v\right)}{\underbrace{\left[\begin{array}{cc} \left(x-x_{0}\right) & v \end{array}\right]}}\underset{\theta_{l}}{\underbrace{\left[\begin{array}{c} k_{l}\\ b_{l} \end{array}\right]}}=F_{e} \end{array}$$

and

(16) $$\begin{array}{c} \underset{Y_{\mathrm{nl}}\left(x,v\right)}{\underbrace{\left[\begin{array}{cc} {\left(x-x_{0}\right)}^{3} & v \end{array}\right]}}\underset{\theta_{\mathrm{nl}}}{\underbrace{\left[\begin{array}{c} k_{\mathrm{nl}}\\ b_{\mathrm{nl}} \end{array}\right]}}=F_{e}\;, \end{array}$$

respectively. These parametrized models can be employed to estimate the coefficients $k_{l}$, $b_{l}$, $k_{\mathrm{nl}}$, and $b_{\mathrm{nl}}$, through standard least squares, but employing the estimated end-effector velocities and forces instead of the measured ones, i.e.,

(17) $$\begin{array}{c} \hat{\theta }={\left(Y^{T}\left(x,\hat{v}\right)Y\left(x,\hat{v}\right)\right)}^{-1}Y^{T}\left(x,\hat{v}\right){\hat{F}}_{e}\; \end{array}$$

where $Y\left(x,\hat{v}\right)=Y_{l}\left(x,\hat{v}\right)$ and $\hat{\theta }={\hat{\theta }}_{l}$ for model (15), and $Y\left(x,\hat{v}\right)=Y_{\mathrm{nl}}\left(x,\hat{v}\right)$ and $\hat{\theta }={\hat{\theta }}_{\mathrm{nl}}$ for model (16).

It is well known that the estimation (17) is more accurate, as [31] (p. 12): (i) the noise-to-signal ratio is low; (ii) the number of samples grows; and (iii) the input power is high. These conditions mean that, to obtain a better estimation of the parameters θ, the larger the forces to be applied are, the better, with a spectrum in all frequencies (i.e., white noise), along with the longest possible experiment duration. Nevertheless, for the palpation application intended in this paper, the mentioned conditions are unrealistic. Furthermore, for a less invasive procedure, it is convenient to have the lowest possible applied force, with the lowest number of frequencies and the shortest possible experiment duration. Thus, a trade-off must be made between these two contradictory requirements. The input power condition can be satisfied by taking into account that for estimation *p* parameters, the number of required frequencies must be at least p/2. Therefore, for estimating the parameters θ in both models (15) and (16), a sinusoidal force signal $F_{e}$ with at least one frequency should be sufficient. Notice, however, that such frequencies must be carefully chosen to be within the bandwidth of the system, which is affected by the robot actuators’ maximum speed, sampling time, and so forth. Regarding the magnitude of the applied force, it must be the largest possible without causing any damage to the tissue. Finally, the experiment duration should be the shortest possible to have consistent estimation values of $\hat{\theta }$ in comparison with longer-duration experiments.

### 2.4. Closed-Loop Force Control

Since the magnitude of the applied force over the tissue is critical for the intended application, a closed-loop force control must be employed. To perform such force control, first, define a desired force profile $F_{d}=F_{d}\left(t\right)$, and set the following feedforward plus integral force controller (alternatively, a Proportional–Integral (PI) controller can be employed as well, as defined in [25] (p. 66)), i.e.,

(18) $$\begin{array}{c} \tau =J^{T}\left(q_{1}\right)J_{\phi x}^{T}\left(F_{d}-k_{Fi}\int_{t_{0}}^{t}\;\left({\hat{F}}_{e}-F_{d}\right)\;d\vartheta \right)\;, \end{array}$$

where $J_{\phi x}$ is the tissue surface normal vector in robot base-coordinates.

### 2.5. Tissue Classification

Suppose that we have *r* different tissues that can be modeled by (3) and (4). An estimation of the model parameters can be obtained by (17) for a single experiment. Now, suppose that several experiments are carried out and a mean $\mu_{j}$ and a standard deviation $\sigma_{j}$ are obtained for a particular parameter of tissue *j*. Let us assume that the distribution of the parameter estimated value is Gaussian, for which the probability of the tissue to belong to a particular class *j*, given an estimated parameter $a_{i}$, can be calculated as

(19) $$\begin{array}{c} P\left(a_{i}|C=c_{j}\right)=\left(1/\sqrt{2\pi \sigma_{j}}\right)e^{-{\left(a_{i}-\mu_{j}\right)}^{2}/\left(2\sigma_{j}^{2}\right)}\;, \end{array}$$

where $a_{i}$ can be any estimated coefficient of models (15) and (16). From the probability calculation in (19) and assuming that all tissues are equally probable, one can construct a Bayesian Classifier (BC) [32] (p. 45) as

(20) $$\begin{array}{c} C=\arg_{C}\left[\max_{j}\left[P\left(C=c_{j}|a_{i}\right)=P\left(a_{i}|C=c_{j}\right)\right]\right]\;. \end{array}$$

Moreover, by assuming conditional independence, *n* of these parameters can be combined to obtain better results by introducing the Simple Bayesian Classifier (SBC)

(21) $$\begin{array}{c} C=\arg_{C}[\max_{j}[P\left(C=c_{j}|a_{1},\ldots ,a_{n}\right)=\prod_{i}^{n}P\left(a_{i}|C=c_{j}\right)]]\;. \end{array}$$

## 3. Results

**Figure 1:**
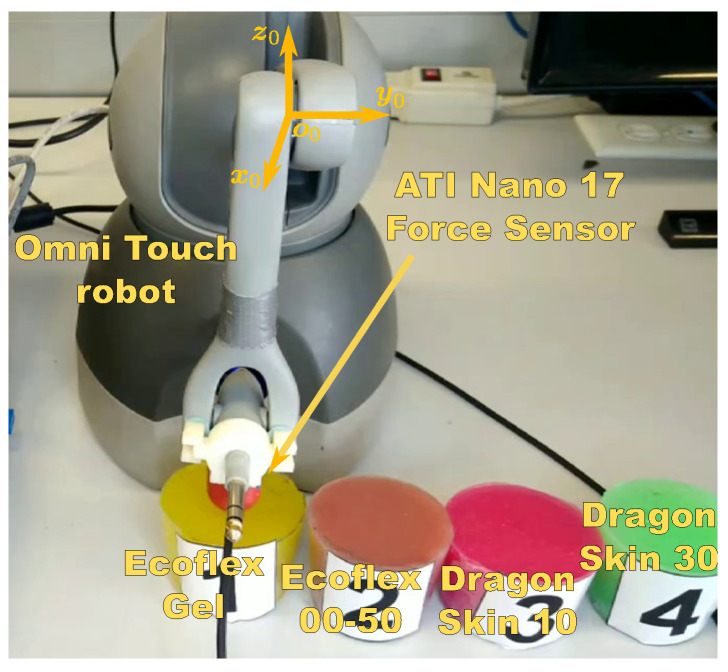
Experimental setup: *3D Systems Omni Touch* robot, *ATI Nano 17* force sensor (only for validation), and the silicone samples *Ecoflex Gel*, *Ecoflex 00-50*, *Dragon Skin 10*, and *Dragon Skin 30* silicone samples.

The experimental setup shown in Figure 1 was set to validate the proposed approach of Section 2. It consists of a *3D Systems Omni Touch* manipulator of six degrees of freedom, from which only the first three are actuated, a six-axes *ATI Nano 17* force sensor (only for validation) and four silicon rubbers with different mechanical properties to represent the tissues: *Ecoflex Gel*, *Ecoflex 00-50*, *Dragon Skin 10*, and *Dragon Skin 30*.

The robot manipulator is programmed and controlled by a *Visual C++* application running on a *PC* with a *Windows Operating System*. The closed-loop sample-time is 2ms, including the force sensor acquisition, robot measurement, and the observer and controller computations. The experiment consists of following a desired force profile over the tissue along the vertical axis, i.e., $F_{d}={\left[\begin{array}{ccc} 0 & 0 & F_{d} \end{array}\right]}^{T}$. As explained in Section 2, the force exerted over the tissue must be carefully chosen to prevent any damage, while still having a relatively low noise-to-signal ratio. For such reasons, the desired force was chosen as

(22) $$\begin{array}{c} F_{d}\left(t\right)=0.9-0.3\cos\left(\pi t\right)+0.2\sin\left(0.3\pi t\right)\;. \end{array}$$

**Figure 2:**
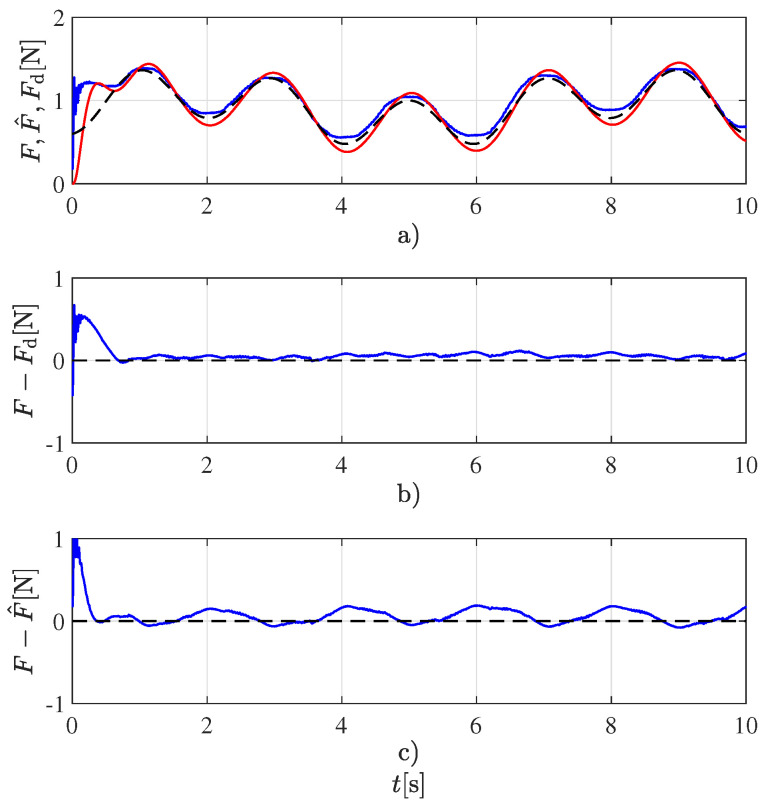
Force tracking and estimation for the Ecoflex 00-50 sample. (**a**) Forces: desired (- - -), measured (—), and estimated (—). (**b**) Force tracking error. (**c**) Force estimation error.

Although a single frequency is sufficient to estimate the two coefficients in each model (3) or (4), the more frequencies there are, the better the excitation of the system. For such reason, a signal with two frequencies was chosen instead of a single frequency signal. It was experimentally determined that increasing the number of frequencies beyond the two employed has no further advantage. This desired force profile, along with the estimated force and the measured one employed for validating the estimated one, are shown in Figure 2. This figure also displays the force tracking error, i.e., the difference between the desired and measured forces and the force estimation error, i.e., the difference between the estimated and measured forces. Although only the experiment for the *Ecoflex 00-50* sample is shown, very similar results can be obtained for the rest of the samples.

**Figure 3:**
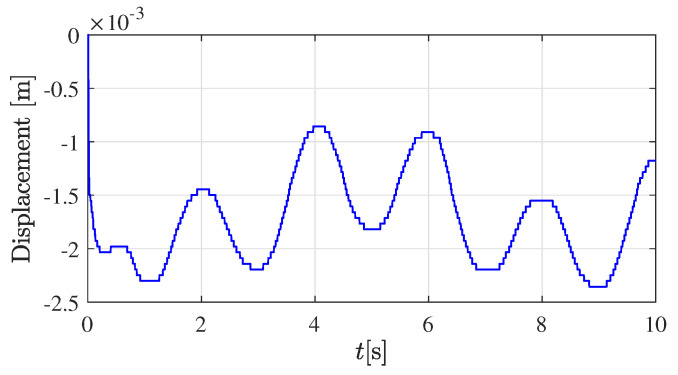
Measured displacement of the tissue for the Eco Flex 00-50 sample.

The corresponding position of the robot’s tip in the vertical direction ($z_{0}$-axis in robot base-coordinates) is displayed in Figure 3. Approximately the first second of the experiment corresponds to the transient response of the closed-loop controller and observer; therefore, it is discarded for the parameters estimator (17).

As mentioned in Section 2, an accurate model of the employed manipulator is required to obtain reliable estimations of the robot velocity and the contact forces. The dynamic model for the three degrees of freedom configuration of the Omni Touch robot employed in this work was taken from [33]. For the sake of completeness of this manuscript, the mentioned model can be found in Appendix A. The robot parameters were obtained by an open-loop free-motion experiment and a standard least squares procedure. In turn, the gains chosen for the observer (8)–(10) were $K_{1}=\mathrm{diag}\left(60,60,60\right)$, $K_{2}=\mathrm{diag}\left(1200,1200,1200\right)$, and $K_{3}=\mathrm{diag}\left(8000,8000,8000\right)$, whereas the force controller gain in (18) was set to $k_{Fi}=5$.

As mentioned in Section 2, the experiment duration must be the shortest possible without implying a significant variation on the estimated parameter mean values. Experimentally, it was found that there is no significant difference in the mean estimation values for experiments with duration $t_{d}>2$ s. Therefore, a set of 32 experiments of duration $t_{d}=2$ s with the force profile given by (22) was carried out. The mean values and the standard deviations for both models (3) and (4) and for each of the four rubber samples are presented in Table 1 and Table 2, respectively. With the aim of validating the estimated coefficients with respect to ground truth, we additionally performed the estimation of k1, b1, knl, and bnl using the real forces measured with the ATI Nano force sensor. A series of four pairwise *T*-Tests applied over the estimated coefficients, comparing the ones obtained with the real versus the estimated contact forces did not reveal any significant differences for any of the four coefficients. Meaning that the proposed force-sensorless identification approach is reliable and valid.

**Figure 4:**
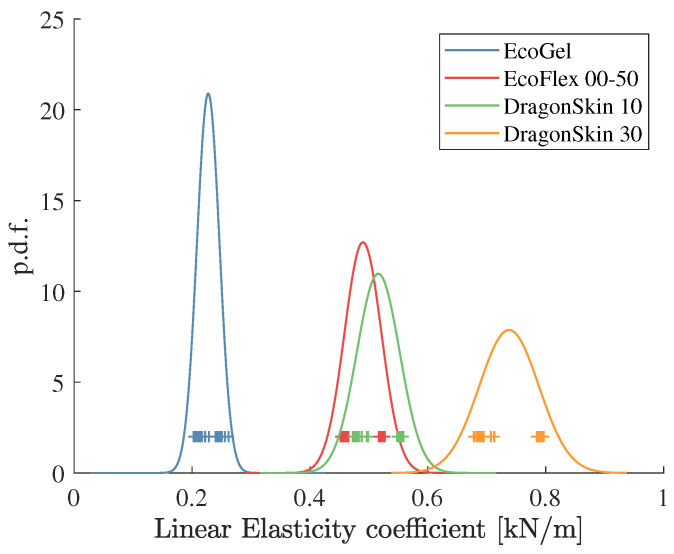
Estimation of the linear elasticity coefficients $k_{l}$ in model (3) for the four different rubber samples and their normal probability density functions.

From these two tables, it can be seen that the elasticity coefficients $k_{l}$ and $k_{\mathrm{nl}}$ posses more information to distinguish between the different samples, i.e., these coefficients are *dominant* over $b_{l}$ and $b_{\mathrm{nl}}$ in models (3) and (4), and thus can be employed for the Bayesian classifier (BC) in (20). A set of another 32 experiments were carried out, considering the estimated linear elasticity coefficient as the input for the BC, i.e., $a_{1}={\hat{k}}_{l}$. The estimated coefficients as well as the probability density functions (pdf) obtained from Table 1 and Table 2 are shown in Figure 4.

From this figure, it can be seen that the *Ecoflex Gel* and the *Dragon Skin 30* samples can be easily distinguished from the other two samples. However, the pdfs of the *Ecoflex 00-50* and the *Dragon Skin 10* have an important intersection area. In fact, these two materials have a very similar stiffness when manually testing them. The percentage of correct classifications (true positives) for all the material samples is shown in Table 3.

**Figure 5:**
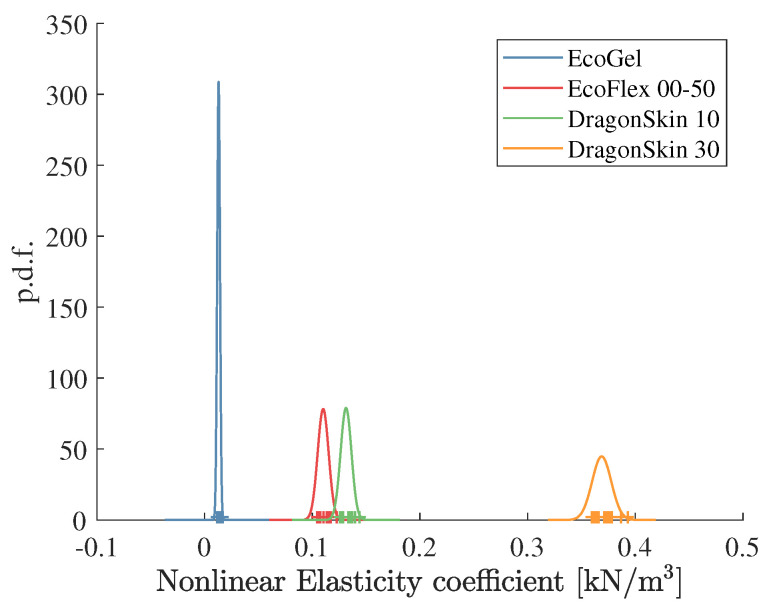
Estimation of the nonlinear elasticity coefficients $k_{\mathrm{nl}}$ in model (4) for the four different rubber samples and their normal probability density functions.

A better classification can be obtained by considering the nonlinear elasticity coefficient $k_{\mathrm{nl}}$ as the input for the BC. The pdfs obtained from Table 1 and Table 2 for this case, along with the outcomes of a new set of 32 experiments, are shown in Figure 5.

When considering the nonlinear spring coefficient ${\hat{k}}_{\mathrm{nl}}$ in (4) as the classifier input, the percentage of correct classifications improves, as displayed in Table 4.

Finally, if instead of a single estimated parameter, a combination of ${\hat{k}}_{l}$ and ${\hat{k}}_{\mathrm{nl}}$ is used in the SBC defined in (21), the classifier always detects the correct rubber sample, as shown in Table 5.

In summary, the experiments presented in this section consisted of the following parts. (1) Each silicone sample is put under the robot tip. (2) The robot exerts the force profile given by (22) by applying the force control (18) in the vertical direction over the tissue. (3) This experiment is repeated several times for each sample to obtain the statistical parameters, i.e., mean and standard deviation. (4) Now, each sample is put randomly below the robot tip. (5) By applying the same force profile and control given by (22) and (18), the parameter estimator and the Bayesian classifier (20), the algorithm returns both the estimated parameters and the tissue classification. An accompanying video where the experiment corresponding to the results of Table 5 is shown can be found at https://youtu.be/tAgwVmYLZp8 (accessed on 7 September 2022).

## 4. Discussion

The results presented in Section 3 show that an approximate estimation of biomechanical parameters can be successfully carried out by employing the robot velocities and contact forces obtained by the Finite-Time Observer (8)–(10) instead of directly measuring them. Although no perfect force reconstruction is possible due to the inherent model inaccuracies and unmodeled external disturbances, the estimation error, as well as the force tracking error, remained relatively low for the magnitude of the applied forces.

The parameters estimated by the method proposed in this work can be employed not only for classification purposes but for simulation of tissues, mathematical analysis of the interaction, haptic interfaces, and so on. For the sake of the proposed application, only the two models (3) and (4) were considered in this work, but any linearly parameterizable model can be employed instead without further modifications.

As already reported in the literature, the linear elasticity coefficient ${\hat{k}}_{l}$ in (3) can be employed as a parameter for classification purposes with relatively good results when the mechanical properties of the involved tissues are very different (e.g., a healthy brain tissue versus a brain tumor, which is approximately 10 times more rigid than the healthy tissue). However, for tissues with similar stiffness the linear elasticity coefficient is no longer accurate to be used as a classifier parameter. In this work, we have found that the nonlinear elasticity coefficient ${\hat{k}}_{\mathrm{nl}}$ in (4) serves as a better input to be used in a BC. Furthermore, if both estimated elasticity coefficients are combined in a SBC given in (21), the tissue classification becomes even more accurate.

Although the parameter estimation given by (17) is, strictly speaking, carried out *offline*, since the experiment duration was carefully chosen to be the shortest possible time (2s for the results presented in Section 3), the acquired data are relatively small and thus the parameters are obtained almost instantaneously in a practical scenario. The proposed method can be directly employed in a teleoperation scheme, in which the physician would not only be able to feel the patient’s tissue due the availability of the estimated force to be employed as a feedback to the operator, but they could “ask” if a particular zone of interest has different mechanical properties with respect to a previously explored one.

Finally, the authors are aware that the experimental study presented in this work is far from being of a practical application. This is mainly due to the limitations of the robot employed for the experiments, for which only the first three of its six degrees of freedom are actuated. Notice however, that the objective of the experiments was to validate the viability of the proposed approach.

## 5. Conclusions

The method proposed in this work is suitable for identifying biomechanical parameters of soft tissues without the need for a force sensor nor direct velocity measurements. To avoid any damage to the tissue, a closed-loop force control is also proposed, which is in turn based on the estimated forces. The approximate parameters estimated by the proposed algorithm can be employed for a realistic simulation of the studied tissues, mathematical analysis of the interaction, and so on. In addition, the estimated parameters can be employed in a real-time procedure for the classification of tissues with different mechanical properties, which can potentially help the physicians to detect abnormalities such as tumors. Moreover, in this work, we have found that a nonlinear viscoelastic model can outperform the results obtained with the commonly employed linear model when the tissues have very similar mechanical characteristics. Finally, if these two models are combined, the classification is further improved.

As for future work, there remains the need to include the approach proposed in this work in a teleoperation scheme, aiming towards its application in a real scenario. The study of other models to represent the tissue mechanics will be explored as well. The application of the proposed scheme for other procedures such as needle insertion will be also investigated.

## Figures and Tables

**Table 1:** Mean values μ of the estimated parameters.

Parameter	${\hat{k}}_{l}\;\left[N/m\right]$	${\hat{b}}_{l}\;\left[Ns/m\right]$	${\hat{k}}_{\mathrm{nl}}\;\left[N/m^{3}\right]$	${\hat{b}}_{\mathrm{nl}}\;\left[Ns/m\right]$
*Ecoflex Gel*	0.2274	0.0047	0.013	$2.02\times 10^{-9}$
*Ecoflex 00-50*	0.4899	$2.81\times 10^{-4}$	0.1101	$4.45\times 10^{-9}$
*Dragon Skin 10*	0.5158	$5.82\times 10^{-5}$	0.1313	$3.55\times 10^{-9}$
*Dragon Skin 30*	0.7377	$6\times 10^{-4}$	0.3688	$3.17\times 10^{-9}$

**Table 2:** Standard deviations σ of the estimated parameters.

Parameter	${\hat{k}}_{l}\;\left[N/m\right]$	${\hat{b}}_{l}\;\left[Ns/m\right]$	${\hat{k}}_{\mathrm{nl}}\;\left[N/m^{3}\right]$	${\hat{b}}_{\mathrm{nl}}\;\left[Ns/m\right]$
*Ecoflex Gel*	0.0191	0.0016	0.0013	$2.11\times 10^{-9}$
*Ecoflex 00-50*	0.0314	$7.82\times 10^{-4}$	0.0051	$6\times 10^{-9}$
*Dragon Skin 10*	0.0364	$4.45\times 10^{-4}$	0.0051	$3.8\times 10^{-9}$
*Dragon Skin 30*	0.0507	$3.17\times 10^{-4}$	0.0089	$3.14\times 10^{-9}$

**Table 3:** Percentage of correct classifications when considering ${\hat{k}}_{l}$ as the BC input.

**Material**	*Ecoflex Gel*	*Ecoflex 00-50*	*Dragon Skin 10*	*Dragon Skin 30*
% Correct class.	100	50	50	100

**Table 4:** Percentage of correct classifications when considering ${\hat{k}}_{\mathrm{nl}}$ as the BC input.

**Material**	*Ecoflex Gel*	*Ecoflex 00-50*	*Dragon Skin 10*	*Dragon Skin 30*
% Correct class.	100	96.88	100	100

**Table 5:** Percentage of correct classifications when considering ${\hat{k}}_{l}$ and ${\hat{k}}_{\mathrm{nl}}$ for the SBC.

**Material**	*Ecoflex Gel*	*Ecoflex 00-50*	*Dragon Skin 10*	*Dragon Skin 30*
% Correct class.	100	100	100	100

## Data Availability

Not applicable.

## Abbreviations

The following abbreviations are used in this manuscript:

FTO	Finite-Time Observer
BC	Bayesian Classifier
SBC	Simple Bayesian Classifier

## Appendix A. Dyamic Model and Parameters for the Touch Manipulator

The dynamic model of the three-degrees-of-freedom configuration of the touch manipulator is taken from [33] (p. 371), where it is described in detail. The matrices of model (1) are parametrized as

(A1) $$\begin{array}{cc} H(q) & =\left[\begin{array}{ccc} c_{2}^{2}\pi_{1}+c_{2}c_{23}\pi_{2}+s_{23}^{2}\pi_{3} & 0 & 0\\ 0 & \pi_{1}+2c_{3}\pi_{2}+\pi_{3} & c_{3}\pi_{2}+\pi_{3}\\ 0 & c_{3}\pi_{2}+\pi_{3} & \pi_{3} \end{array}\right] \end{array}$$

(A2) $$\begin{array}{cc} g(q) & =\left[\begin{array}{c} 0\\ g\left(c_{2}\pi_{7}+c_{23}\pi_{8}\right)\\ gc_{23}\pi_{8} \end{array}\right]\;, \end{array}$$

whereas the components of the $C(q,\dot{q})$ matrix are

$$\begin{array}{cc} c_{e11} & =-c_{2}s_{2}{\dot{q}}_{2}\pi_{1}-\frac{1}{2}\left(c_{2}s_{23}\left({\dot{q}}_{2}+{\dot{q}}_{3}\right)+s_{2}c_{23}{\dot{q}}_{2}\right)\pi_{2}+c_{23}s_{23}\left({\dot{q}}_{2}+{\dot{q}}_{3}\right)\pi_{3}\\ c_{e12} & =-c_{2}s_{2}{\dot{q}}_{1}\pi_{1}-\frac{1}{2}\left(s_{2}c_{23}+c_{2}s_{23}\right){\dot{q}}_{1}\pi_{2}+s_{23}c_{23}{\dot{q}}_{1}\pi_{3}\\ c_{e13} & =-\frac{1}{2}c_{2}s_{23}{\dot{q}}_{1}\pi_{2}+c_{23}s_{23}{\dot{q}}_{1}\pi_{3}\\ c_{e21} & =c_{2}s_{2}{\dot{q}}_{1}\pi_{1}+\frac{1}{2}\left(s_{2}c_{23}+c_{2}s_{23}\right){\dot{q}}_{1}\pi_{2}-s_{23}c_{23}{\dot{q}}_{1}\pi_{3}\\ c_{e22} & =-s_{3}{\dot{q}}_{3}\pi_{2}\\ c_{e23} & =-s_{3}\left({\dot{q}}_{2}+{\dot{q}}_{3}\right)\pi_{2}\\ c_{e31} & =\frac{1}{2}c_{2}s_{23}{\dot{q}}_{1}\pi_{2}-c_{23}s_{23}{\dot{q}}_{1}\pi_{3}\\ c_{e32} & =s_{3}{\dot{q}}_{2}\pi_{2}\\ c_{e33} & =0\;. \end{array}$$

In turn, the friction terms were considered only viscous, i.e.,

$$\begin{array}{c} \tau_{f}\left(\dot{q}\right)=\left[\begin{array}{c} \pi_{4}{\dot{q}}_{1}\\ \pi_{5}{\dot{q}}_{2}\\ \pi_{6}{\dot{q}}_{3} \end{array}\right]\;. \end{array}$$

Due the force sensor attached to the tip of the manipulator, the parameters $\pi_{i}$ no longer correspond to the ones reported in [33] (p. 371), so a new identification was carried out, giving as a result the set of parameters displayed in Table A1.

**Table A1:** Parameters estimation for the 3 DOF configuration.

Parameter	Estimated Value
$\pi_{1}$	0.00027632
$\pi_{2}$	0.00056422
$\pi_{3}$	0.00084797
$\pi_{4}$	0.01706838
$\pi_{5}$	0.01920879
$\pi_{6}$	0.01622868
$\pi_{7}$	0.00357954
$\pi_{8}$	0.00551499
